# Virtual and augmented reality in biomedical engineering

**DOI:** 10.1186/s12938-023-01138-3

**Published:** 2023-07-31

**Authors:** Aya Taghian, Mohammed Abo-Zahhad, Mohammed S. Sayed, Ahmed H. Abd El-Malek

**Affiliations:** 1 Department of Electronics and Communications Engineering, Egypt–Japan University of Science and Technology, New Borg El-Arab City, Alexandria Egypt; 2 Department of Electrical Engineering, Assiut University, Assiut, Egypt; 3 Department of Electronics and Communications Engineering, Zagazig University, Zagazig, Ash Sharqia Egypt

**Keywords:** Augmented reality, Biomedical context, AR surgery, AR in education, Virtual reality

## Abstract

**Background:**

In the future, extended reality technology will be widely used. People will be led to utilize virtual reality (VR) and augmented reality (AR) technologies in their daily lives, hobbies, numerous types of entertainment, and employment. Medical augmented reality has evolved with applications ranging from medical education to picture-guided surgery. Moreover, a bulk of research is focused on clinical applications, with the majority of research devoted to surgery or intervention, followed by rehabilitation and treatment applications. Numerous studies have also looked into the use of augmented reality in medical education and training.

**Methods:**

Using the databases Semantic Scholar, Web of Science, Scopus, IEEE Xplore, and ScienceDirect, a scoping review was conducted in accordance with the Preferred Reporting Items for Systematic Reviews and Meta-Analyses (PRISMA) criteria. To find other articles, a manual search was also carried out in Google Scholar. This study presents studies carried out over the previous 14 years (from 2009 to 2023) in detail. We classify this area of study into the following categories: (1) AR and VR in surgery, which is presented in the following subsections: subsection A: MR in neurosurgery; subsection B: spine surgery; subsection C: oral and maxillofacial surgery; and subsection D: AR-enhanced human-robot interaction; (2) AR and VR in medical education presented in the following subsections; subsection A: medical training; subsection B: schools and curriculum; subsection C: XR in Biomedicine; (3) AR and VR for rehabilitation presented in the following subsections; subsection A: stroke rehabilitation during COVID-19; subsection B: cancer and VR, and (4) Millimeter-wave and MIMO systems for AR and VR.

**Results:**

In total, 77 publications were selected based on the inclusion criteria. Four distinct AR and/or VR applications groups could be differentiated: AR and VR in surgery (*N* = 21), VR and AR in Medical Education (*N* = 30), AR and VR for Rehabilitation (*N* = 15), and Millimeter-Wave and MIMO Systems for AR and VR (*N* = 7), where *N* is number of cited studies. We found that the majority of research is devoted to medical training and education, with surgical or interventional applications coming in second. The research is mostly focused on rehabilitation, therapy, and clinical applications. Moreover, the application of XR in MIMO has been the subject of numerous research.

**Conclusion:**

Examples of these diverse fields of applications are displayed in this review as follows: (1) augmented reality and virtual reality in surgery; (2) augmented reality and virtual reality in medical education; (3) augmented reality and virtual reality for rehabilitation; and (4) millimeter-wave and MIMO systems for augmented reality and virtual reality.

## Introduction

**Fig. 1:**
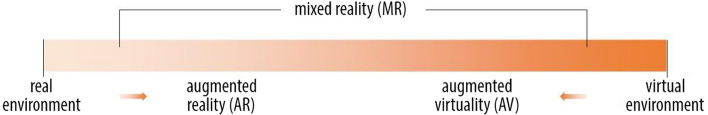
MR, AR, and VR [4]

The phrase “extended reality” (XR) refers to all virtual reality (VR), augmented reality (AR), or mixed reality (MR) technology (see Fig. 1), which are technologies that have the potential to revolutionize the way we work. In addition, they help us to interact with each other and the environment, and the way we experience and feel things. These technologies have become very popular over the last few years due to the numerous recent evolutions of the technologies. Not only because they make up the architecture of XR systems, of which photonics is one of the pillars, but also due to society truly accepting them, which is a relatively new phenomenon. Each of these concepts for XR, MR, AR, or VR has a unique quirk: VR captivates the user in a completely virtual world created by optical systems and monitors, often incorporated into a head-mounted system. AR refers to an interactive experience of a real environment with information generated by optical systems and is similar to classic glasses, and MR is merging AR and VR solutions like Microsoft HoloLens [1].

Paul Milgram established the notion of a reality–virtual continuum in 1994 [2]. However, because of technological restrictions in that era, little progress was made until the early 2010 s, when fresh technological advancements permitted the AR/VR machine to be revitalized. Since then, we have seen an increase in investment opportunities in these innovations, as well as extensive client interest in the gaming universe and, more notably, the “metaverse,” which is drawing the world’s largest IT businesses (Meta, Google, Microsoft, Apple, etc.).

AR technologies for the consumer market are now mature for many possible domains of application. There is a high desire for alternatives that can improve the existing clinical practice in the healthcare industry, as seen by the growing number of publications on AR for surgery, medicine, and rehabilitative services.

The goal of this special matter is to provide engineers, computer scientists, and end-users with an outline of the potential of AR technologies to foster the development of useful applications in the near future. Moreover, to direct scientific studies toward conquering the technological and societal problems that still exist among today’s devices and the most famous modalities for augmenting the visual sensation with computer-generated aspects [2, 3]. Even while AR devices and applications have almost all focused on enhancing the eyesight so far, the augmentation of other sensations has yet to achieve the same general adoption.

From a technical aspect, it is vital to note that video-based tracking may be accomplished with a marker-free tracking strategy using feature recognition on the sufferer. Overall, deciding where a single AR software should be positioned inside the reality–virtuality is tricky. This is particularly true in medical AR, where a great deal of patient-specific data and photos is accessible, and it can be difficult to tell if digital material displayed on a display is real or virtual. There are several methods for acquiring and combining real and virtual data in a way that is beneficial to the user. The visualization of both VR and AR modalities might be an additional benefit for some projects [4].

This paper presents A recent review of the latest AR and VR applications in the biomedical engineering context over the past few years. Furthermore, it provides a wider scope of applications with a modern classification. In this work, we categorize this research area according to the following dimensions: (1) AR and VR in surgery, which is presented in the following subsections: subsection A: MR is neurosurgery; subsection B: spine surgery; subsection C: oral and maxillofacial surgery; and subsection D: AR-enhanced human-robot interaction; (2) AR and VR in medical education; (3) AR and VR for rehabilitation presented in the following subsections; subsection A: stroke rehabilitation during COVID-19; subsection B: cancer and VR, and (4) Millimeter-wave and MIMO systems for AR and VR. To direct and educate future research in AR and VR in biomedical engineering, we identify significant problems and possibilities.

Furthermore, we describe the methodology used to conduct a scoping review of the literature in the field of AR/VR in healthcare. To do so, we utilized several databases, including Semantic Scholar, Web of Science, Scopus, IEEE Xplore, and ScienceDirect, and conducted a manual search in Google Scholar to find relevant articles. We followed the Preferred Reporting Items for Systematic Reviews and Meta-Analyses (PRISMA) criteria to ensure that the review was comprehensive and transparent. The study covers a period of 14 years, from 2009 to 2023, and provides a detailed analysis of the studies conducted during this period.

Finally, by categorizing the studies into the previously mentioned four main areas, we provide a comprehensive overview of the research conducted in the field of AR/VR in healthcare. This categorization also allows for a more in-depth analysis of the research, making it easier for readers to identify relevant studies in their area of interest. Overall, the methodology used in this study ensures that the scoping review is rigorous and comprehensive, providing valuable insights into the use of AR/VR in healthcare.

## AR and VR in surgery

### MR in neurosurgery

Every 2 decades or so, technological innovation in neurosurgery makes a giant leap ahead. The first commercial computed tomography (CT) scans were accessible in the 1970s, and the microscope entered the neurosurgery operating room in the late 1950s. The use of VR in neurosurgery began in the 1990s. The next iteration of the jump might be represented by MR. However, unlike the microscope, which changed operating visualization, and CT scans, which replaced pneumoencephalograms for diagnosis, MR has a wide range of neurosurgical uses.

As pilots employed VR for flight simulation to prevent causing damage to planes and causing physical injury to the student, neurosurgeons began to use VR technology for teaching, because it allowed for “do-overs” without endangering patients. The three-dimensionality of complicated topography, the terrain for the former, and the anatomy of the skull and spine for the latter are what connect a flight route with a surgical approach [5]. Nevertheless, since its inception in the development of neurosurgical skill and experience, the use of MR has evolved to include not just the assessment of the learner, but also surgical planning, operative visualization, and significantly transforming the nature of neuronavigation. The numerous uses of MR also pervade much of modern-day neurosurgery, as the articles in this issue reveal, from vascular to oncological and spinal operations.

Researchers like Tagaytayan et al. in [6] examined the application of AR in neurosurgery for simulation and training in their paper. Their findings indicate that AR has a lot of promise in neurosurgery and training. AR, according to the investigators, allows for precise pre-operative planning as well as excellent intraoperative navigation. They further praise AR’s ability to provide a safe setting for realistic neurosurgical operation training. The researchers also discuss how AR may be employed in nursing and other disciplines of healthcare. They also reaffirm neurosurgeons’ strong recommendations to adopt AR as a supplement to existing technologies in the operating room to match their present workflow.

From remote proctoring of difficult operations to training young neurosurgeon trainees throughout the world, MR will likely play a big role in global neurosurgery in the future. A surgery practiced, executed, and recorded in VR in one part of the world can be repeated for a patient in another part of the world without time zone limits when combined with robots. Visualizing data from tens of thousands of people was combined with artificial intelligence. It is possible to get a list of foreign patients with a certain uncommon tumor. gathered, transformed into virtual models, and put to the test in VR. Various surgical exposures are assessed to determine the optimal surgical choice. Hopefully, as the cost of equipment falls and Internet connectivity improves, MR technologies will be more practical for patients all around the world [7].

In neurosurgery, XR has been shown to be beneficial in pre-operative planning and multimodal neuronavigation. Furthermore, the papers included in the review revealed that XR can help with neurosurgery and neurosurgical training. This section discussed the vast potential of VR in neurosurgery, which goes beyond only planning surgical operations. We need to thoroughly examine AR and VR deployments to fully understand the benefits and limits of a head-mounted display (HMD) and other technologies used during surgery, as well as in all aspects of neurosurgery, to provide meaningful data in the future. The use of XR in neurosurgical teaching is encouraging and merits more investigation.

### Spine surgery

XR implementation in spinal surgery has expanded rapidly over the past decade [8]. Pedicle screw placement, targeted cervical foraminotomy, bone biopsies, osteotomy planning, and percutaneous intervention have all been done with AR in spine surgery. Accurate placement of pedicle screws is a common problem that is critical to the structure’s quality and durability. This is particularly true for minimally invasive procedures, where navigation is more difficult and the anatomy of the posterior spinal column is not commonly accessible.

For pedicle screw placement, the authors of [9] introduced a unique AR-based surgical navigation system. Ultrasound was utilized to connect three-dimensional (3D) anatomic landmarks to CT scans. They then projected the surgical plan onto the live patient. They employed K-wires instead of pedicle screws in their investigation and compared their approach to skin marker tracking, in which proportional soft-tissue deformations impact the linkage of biomarkers and CT images. Their ultrasonic AR technology had a mean targeting error of 3.79 mm and a mean angle error of 4.51 degrees, compared to skin marker tracking, which had mean aiming and angle errors of 5.18 mm and 5.89 degrees, correspondingly. They evaluated the feasibility and accuracy of AR surgical navigation (ARSN) for pedicle screw insertion in three experiments. A surgical navigation system was built in a hybrid OR where the table was connected to a motorized ceiling-mounted C-arm system for 2D-3D imaging as part of their research. Four video cameras were used to track marks on the subject that were set at unexpected times.

ARSN had an average precision of 85%, compared to 64% for free-hand positioning. ARSN without fluoroscopy was found to be better for free-hand thoracic pedicle screw placement. They performed 20 operations in which one orthopedic spine surgeon used the ARSN device to insert 253 pedicle screws without utilizing fluoroscopy. With no significantly misplaced screws and an average screw placement time of 5.2 min, the total accuracy was 94.1%. This highlights AR systems’ enormous potential in the surgery room for open or minimally invasive spine surgery.

Later, Burstorm et al. [10] tested the practicality and efficiency of their radiation-free ARSN system designed for pedicle cannulation on two pig cadavers, based on the work of Elmi Terander et al. Clinical accuracy was 97.4%, relative to 89.3 percent for free-hand insertion and 92.3% for robotic-assisted guidance. Molina et al. [11] explored the accuracy of AR systems to pedicle screw insertion in another proof of concept. A total of 120 pedicle screws were implanted in 5 cadaveric male torsos ranging in size from T6 to L5. Using postprocedural CT scans, two independent neuroradiologists evaluated the Gertzbein scale and its combination with the Heary classification (Heary–Gertzbein scale). In comparison to free-hand placement, computer navigation, and robotic-aided pedicle insertion, the AR guidance system outperformed them on both scales [12, 13].

When compared to the Free-Hand insertion method, the accuracy of the Pedicle Screw Placement was shown to rise in the in vivo, cadaver, and phantom model groups using Augmented Reality Head-Mounted Displays (AR-HMDs). Data on user experience and satisfaction were scarce, but when they were included, a clear benefit for the operation results was noted. Screwing using Robot-Assisted Surgery (RAS) produced similar accuracy results. It is clear that AR-HMDs require benchmarking and quantified situation awareness. Based on assessments of the surgeon’s (the end-users) user happiness, clinical accuracy, and procedure time, the authors provide a system for standardized scoring and visualization of surgical navigation technology. To recap, computer technology has long been used to help with spine surgery, and it is effective for some treatments. AR-HMD navigation is now technologically capable of being used in surgery and is a more inexpensive alternative to RAS. To match the potential of RAS/XR in human procedures, enhancements in ergonomics and accessibility are required [14].

Percutaneous vertebroplasty has also shown promise in AR. Abe et al. [15] developed a virtual protractor with an augmented reality (VPAR) head-mounted AR guiding system to see needle trajectory during vertebroplasty. To correlate the needle trajectory course to the patient’s body, they employed a tracking camera and skin markers. They tested their strategy on 40 phantom spine experiments and five patients. They acquired an angular variation of 0.96 degrees from the anticipated trajectory in their phantom experiments. Five patients with osteoporotic vertebral fractures received VPAR as part of their clinical assessment.

In the axial and sagittal planes, postoperative CT examination revealed average errors of 2.09 degrees and 1.98 degrees, respectively. Yamamoto et al. [16] employed AR for minimally invasive keyhole surgery in both transvertebral anterior cervical foraminotomy and posterior cervical laminoforaminotomy in another use of AR in minimally invasive spine surgery. To construct an AR navigation system, they used O-arm imaging. Both procedures were successful, and patients remained symptom-free for an increase of 20 months following the procedure.

Artificial intelligence (AI) is evolving at a breakneck pace, thanks to recent advances in deep learning approaches. The use of AR and VR in healthcare is gaining traction, and spine surgery is no exception. Low cost, flexible interaction with other technologies, user-friendly functionality, and use in navigation systems are just a few of the unique capabilities and benefits of AR and VR devices, which make them useful in a variety of elements of spine surgery. Despite the use of AR for pedicle screw placement, targeted cervical foraminotomy, bone biopsy, osteotomy design, and percutaneous intervention, AR and VR applications in spine surgery are still restricted [17].

### Oral and maxillofacial surgery

The attractiveness of medical AR stems from the concept of “X-ray” vision, or the capacity to see into objects. In medicine, where an inside picture of the body of the patient is readily accessible through medical imaging, such as X-rays, CT, or magnetic resonance imaging (MRI), this information can be blended into the same physical space as the sufferer to provide in situ vision [18].

Compared to the usual technique of viewing medical imaging data on a separate 2D monitor, this novel in situ visualization paradigm has significant advantages. Physicians no longer need to cognitively translate imaging data to the patient thanks to AR. This is useful in a variety of situations, including physical examinations and treatment planning, but it is most useful during image-guided procedures. In the present practice, navigation data are shown on one or more monitors situated surrounding the surgical site, which is a difficult undertaking in and of itself due to space limits and line-of-sight demands.

**Fig. 2:**
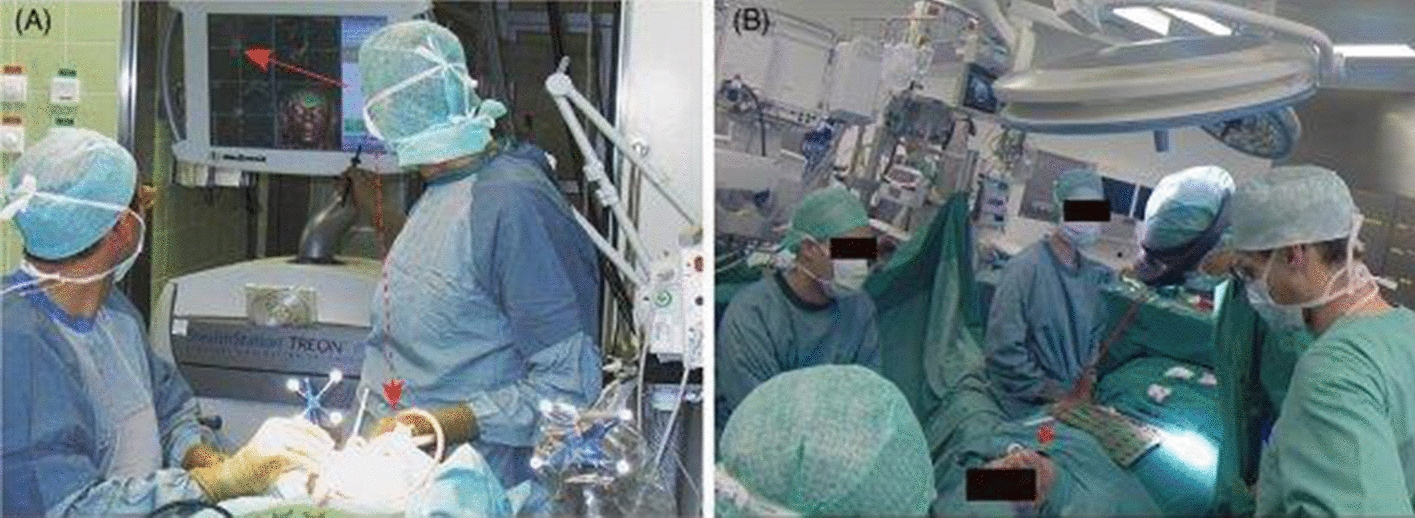
Comparison between traditional image-guided surgery (**A**) and AR-guided surgery (**B**) [18]

The outcome is that the physician is obliged to split his focus between navigation and the patient, all while synchronizing surgical instrument movements. By employing situ visualization, the surgeon’s focus is not diverted, resulting in greater hand–eye coordination and, as a result, increased accuracy and time efficiency. Figure 2 shows a comparison between standard image guiding and AR-based image guidance. The better perception of imaging data in support of the physician’s decision-making process is a second key benefit of medical AR. Modern AR screens allow for the stereoscopic representation of volumetric data, providing a real 3D perspective. This causes perceptual signals like binocular disparity and motion parallax to emerge, resulting in a better spatial knowledge of structures.

Moreover, synchronizing the viewpoints of actual and virtual objects automatically assists in 3D data examination by making the perspective modification more natural.

In 1995, oral and cranio-maxillofacial surgery (OCMS) was one of the first specialties to be studied in the context of medical AR.

This was most likely due to the fact that OCMS is a surgical specialty that deals with complicated anatomy and where image guidance has a lot of promise. The larynx, mouth cavity, and complex neurological system all reside in the oral and cranial area, and if they are destroyed during surgery or therapy, the patient may be unable to talk, swallow, or be severely deformed. Only extremely precise techniques can provide a surgical outcome that is both functional and aesthetically acceptable, while also preserving the patient’s quality of life to the greatest extent feasible. As a result, OCMS implantology, orthognathic surgery, trauma, and cancer have all used AR [18].

In the 1980s, the notion of computer-assisted procedures in the head and neck areas was initially discussed. Surgical navigation systems have been used in several subspecialties of OCMS since then, including trauma surgery, foreign body removal, tumor excision, reconstructive surgery, and orthognathic surgical intervention. The brain, eyes, larynx, and mouth cavity are all housed in the cranio-maxillofacial complex, which contains sensitive architecture and vital organs. Surgical procedures in this area, therefore, have high accuracy requirements to provide a functional and aesthetically ideal treatment for the client. Optical and electromagnetic navigation devices are still widely employed in OCMS clinics in terms of handling technology.

**Fig. 3:**
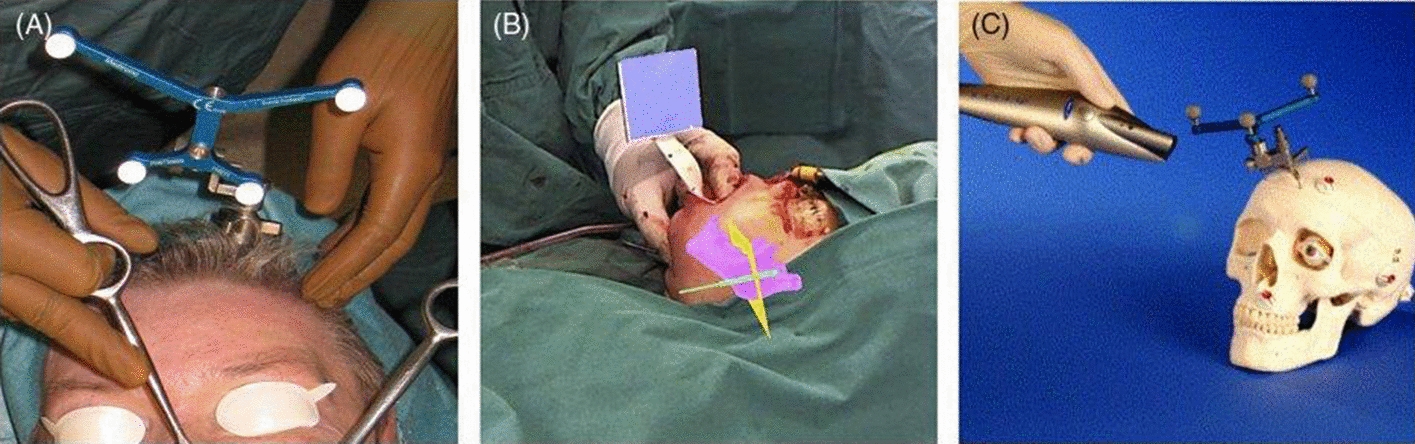
Image-to-patient registration strategies in OCMS. **A** In traditional navigated OCMS, a skull-fixed marker, here using retro-reflective spheres for tracking with an infrared sensor, is used for registration. **B** Alternatively, using patient-specific occlusal splints for non-invasive marker fixation has been proposed. **C** Marker-less registration, for example, using a laser scanner to capture the outer anatomy of the patient has also been suggested. However, to track the movement of the patient, a marker is still required [18]

The advantages of integrated global services (IGS), on the other hand, come at a price. The use of traditional navigation adds to the complexity of a procedure. Image-to-patient registration is the first hurdle. Most guided OCMS procedures need the intrusive attachment of fiducial markers to the skull, as shown in Fig. 3A. Instead, the non-invasive use of an occlusal dental splint to retain reference markers has been proposed (see Fig. 3B), however, because of the moveable temporomandibular joints and the narrow operational field, it is considerably more prone to mistake. Furthermore, because patient-specific dental casts must be created for surgery, preparation and planning time are increased. Following the installation of the markers, specialist pre-operative scans must be obtained, which will be compared to the patient in the OR and utilized for image guiding. Because this procedure needs both time and human resources, it must be scheduled multiple days ahead of time. Simultaneously, CT scanning, the most often used imaging modality in OCMS, increases the patient’s radiation dosage in a vital location. Marker-less approaches, such as employing a laser scanner to digitize the patient’s skin surface, as shown in Fig. 3C, have been considered as viable alternatives, but they need the subject to stay immobile and are thus limited to certain applications.

A further drawback is that IGS systems often display medical imaging and planning data in two dimensions on an external display, whereas anatomical structures and surgical interactions are 3D. This demands the surgeon to divide his concentration between the navigation system and the operation site, as well as complicated hand–eye coordination, which can only be learned via decades of training. By sending imaging data directly in the same reference space as the patient, in situ visualization via AR technology should ease this challenge, increasing the spatial awareness of targeted anatomies and the synchronization of 3D engagements.

While contemporary AR platforms are less costly than traditional robotics and navigation systems, they nevertheless have substantial upfront capital expenses. The ambulatory surgical center and/or surgeon-owned institution find it less tempting because of the disposable expenditures for each case. An intraoperative CT scan is also required by the currently available systems (as opposed to a pre-operative CT scan). If you do not already have intraoperative CT scan technology, your initial capital expenses will skyrocket. Upcoming incarnations of the technology should address problems like eye fatigue and neck strain. AR technology has a promising future. We anticipate that AR, robots, navigation, and endoscopy will all be combined in the future [19].

As procedures are not limited to the above, to develop a state-of-the-art and to offer hints of future prospects, authors in [20–22] identify, catalog, and analyze series describing AR approaches tested in liver, prostate, and hip surgery.

### AR-enhanced human–robot interaction

**Fig. 4:**
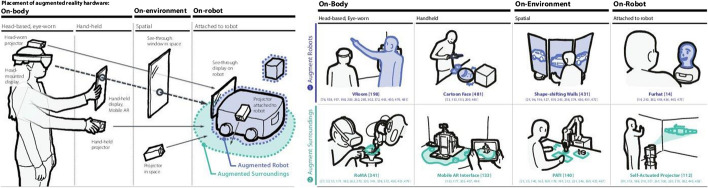
Approaches to augmenting reality in robotics [24]

Human–robot interaction (HRI) and robotic interfaces have benefited from the use of MR. An escalating number of research in HRI and robotics have recently revealed how AR facilitates improved human–robot interactions [23]. Nonetheless, research is frequently focused on individual investigations and essential design techniques, with research topics rarely examined in a systematic manner. Figure 4 depicts many methods of AR in robotics. They are divided into two categories depending on 2D: First, there are ways dependent on where the AR hardware is placed. Three possible sites were proposed: (1) on-body, (2) on-environment, and (3) on-robot. Second, a categorization based on the visual augmentation target location is divided into two categories: (1) augmenting robots and (2) augmenting surroundings.

#### Approach-1

- Augment robots: AR is used to enhance robots by superimposing or anchoring new information on top of them (Fig. 4 Top) [24].
- On-body: The first group uses on-body AR gadgets to augment robotics. HMD or mobile AR interfaces are two options. VRoom, for example, enhances the telepresence robot’s look by superimposing a remote user on top of it. On mobile AR devices, systems showed putting an animated face on a robot to portray an expressive feeling.
- On-environment: This category adds gadgets to robots that are implanted in their surroundings. This strategy frequently employs technologies, such as (1) environment-attached projectors or (2) see-through displays. Drone-SAR, for example, demonstrates how projection mapping may be used to complement the drone itself. This can also include displaying superimposed information on top of robotic interfaces. The superimposed animation of data is also directly reinforced by a shape-shifting wall or portable shape-changing interfaces.
- On-robot: In the third category, robots augment their own looks, which is a first in the field of AR and robotics. For example, a back-projected robot head is used to animate a face, so that the robot may enhance its own face without the use of an exterior AR device. Robot-attached displays, which enhance themselves by utilizing their own bodies as screens, are a widespread technology. Robot-attached monitors, on the other hand, may fit under this group.

#### Approach-2 [24]

- Augment surrounds: Alternatively, AR may be utilized to enhance the robots’ settings. Surroundings in this instance comprise (1) mid-air 3D space, (2) tangible items, or (3) physical environments, such as walls, floors, ceilings, and so on (Fig. 4 Bottom).
- On-body: Related to HMDs, mobile AR gadgets, and portable projectors, this category augments robots’ environments. An expressiveness rendering capacity afforded using 3D graphics and spatial scene comprehension is one feature of HMD or mobile AR devices. Drone Augmented Human Vision, for instance, employs HMD-based AR to modify the look of the wall to operate drones remotely. RoMA uses an HMD to overlay interactive 3D models on a robotic 3D printer [24].
- On-environment: unlike HMDs or mobile devices, the on-environment method makes sharing AR experiences with co-located users considerably easier. Projection modeling or surface displays can be used for augmentation. Touch and Toys, for instance, use a huge surface display to offer more information about the robot’s environment.

The use of projection mapping to highlight or augment surrounding items to indicate the robot’s intentions is under investigation. Even as it allows numerous individuals to share material, the disadvantage of this strategy is that it requires a permanent position owing to installed-equipment requirements, which might also limit flexibility and range of motion in outside circumstances.

- On-robot: In this category, the robots themselves augment the surrounding environment. The use of a robot-attached projector to supplement surrounding physical settings has been highlighted as a typical strategy. A projector coupled to a mobile robot, for example, might be used to create a self-propelled projector for ubiquitous displays. Furthermore, for on-demand displays, Display-Drone projects a picture into the surrounding walls. The fundamental advantage of this technique is that it eliminates the need for on-body or environment-instrumented equipment, allowing for transportable, adaptable, and deployable engagements in a variety of scenarios.

Despite the apparent technological superiority of image-guided interventional robots, the authors looked at the underlying causes of this difference. They mentioned the da Vinci robot and how it achieved commercial success and riding that wave over the past 10 years, and gynecology has emerged as the most popular surgery type with da Vinci. With robotic help, human surgeons are now able to conduct numerous surgeries using minimally invasive techniques. Imaging and telerobotic integration were made possible in costly interventions with profitable results for investors. A pre-surgical-centered approach is instructive in multiple ways, and image-guided interventional robotic systems are also categorized in other ways. Concepts and components of image-guided interventional robotic systems are outlined within the context of larger computer-integrated surgical and interventional systems [25].

## VR and AR in medical education

### Medical training

Modeling and adequate professional training, according to the Institute of Medicine, are the keys to comprising a total for successful collaborative practice [26–29]. The authors are purposely [6] assessing the impact of AR on surgical and clinical outcomes. To accomplish this, they created the ARiSE program (AR in Surgery and Education). ARiSE might be used to create 3D holograms that people can view in real-world settings using AR goggles. For interactivity, the software employs UI buttons and voice commands. This program will let users see human organs on real-world surfaces for diagnostic or training reasons. ARiSE may be used in a variety of settings, including surgery and nursing education.

**Fig. 5:**
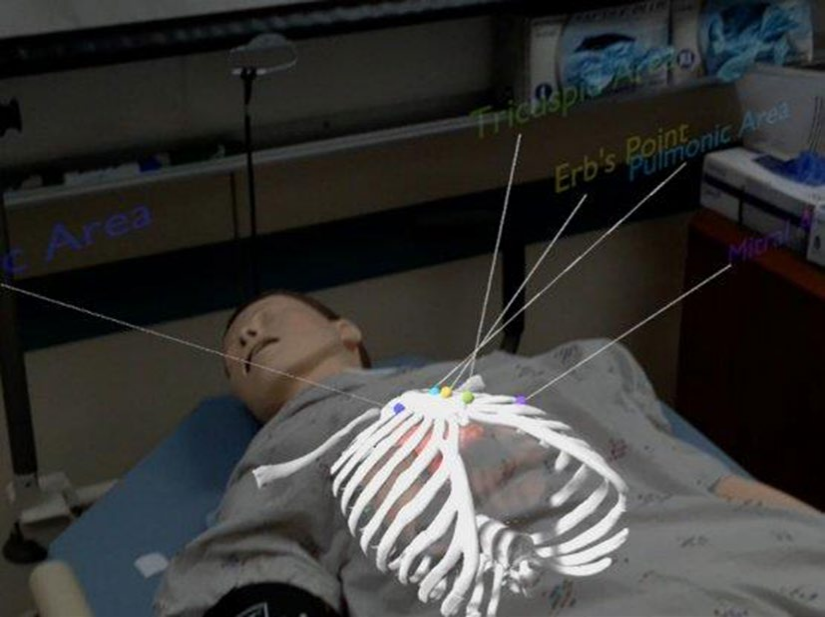
AR being used on a manikin [6]

Learners wearing AR headsets are seen in Fig. 5 with a holographic overlay of generic and animated representations of the heart, lungs, and rib cage. Throughout the training, this would be precisely set to coincide with a manikin or a real participant. Accurate auscultation sites’ anatomical landmarks will also be accessible [6].

**Fig. 6:**
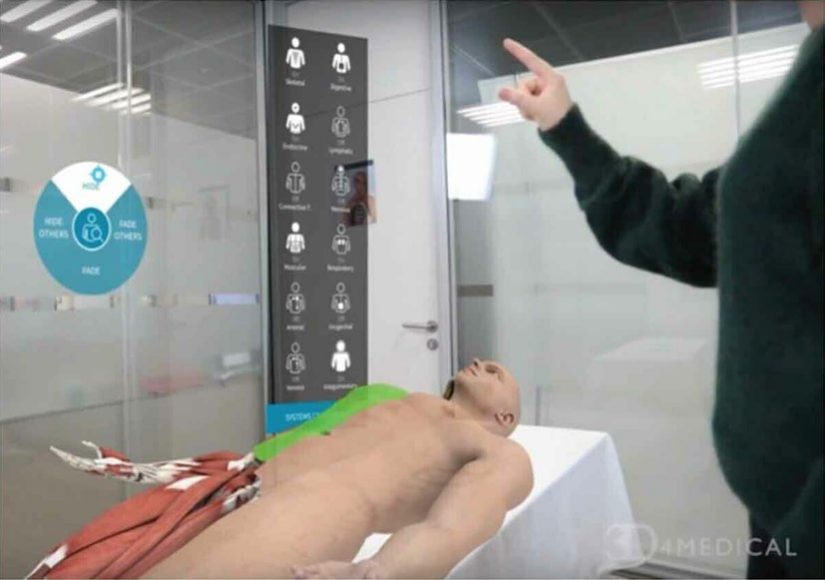
The AR app “HoloHuman” shows a virtual cadaver placed on a real examination table [33]

Medical education entails a tremendous quantity of material on human anatomy and body function [30, 31]. The introduction of a myriad of digital applications, such as ’virtual cadavers,’ has tremendously helped the learning of this material (Fig. 6). AR can increase the way medical learners participate with digital anatomical depictions from all angles, delivering a more interactive environment that eventually enhances understanding and knowledge [32], rather than being accessed via a standard computer mouse, keyboard, and screen.

As shown in Fig. 6, a simulated cadaver is put on a real examination table in the AR software HoloHuman. Through the use of a HoloLens headset, the moderator (pictured) may interact with the model and user interface. Visual storytelling and digital dissection tools facilitate the examination of structures, organs, and systems separately or in combination.

The usage of AR platforms in medical education has developed dramatically from the first use of these platforms in orthopedic illnesses. The expense of building these interactive platforms is one of the biggest difficulties facing the higher education sector [34]. This, along with a scarcity of resources to satisfy the requirements of rising student populations, obstructs their use in medical education. Educators’ main challenge is making digital technology equal and accessible to all pupils. Another critique of AR in education is the restricted technology required, as well as the developing issue of social isolation connected with digital learning [35]. Nonetheless, these new digital platforms have allowed educators to push the boundaries of traditional pedagogies to provide students with student-centric, interactive, and meaningful educational experiences.

In [36], the purpose of this study was to provide an overview of the research and development of a newly created MR and haptic-based dental simulator for tooth preparation and to carry out a preliminary assessment of its face validity. Unidental MR Simulator for tooth preparation, recently researched and created, has good facial validity. By enabling users to change the posture of the patients, it can give them a more realistic skill training experience that is more akin to the actual clinical treatment environment. To improve the simulation experience, the display’s visual representation of the tooth preparation simulation and the teeth’s force feedback need to be improved.

### Schools and curriculum

Also, with improvements in the sector, the use of AR literature in clinical school, which was initially suggested more than a generation later, has been suggested as a crucial next step. Yuen [37] provided an explanation of how AR textbooks will allow learners to immerse themselves in a scenario or circumstance and learn by doing so. Although these articles have steadily been incorporated into education curricula over the decades, their application in higher education for teaching medical services and pharmaceutics topics is still in its early stages [38, 39].

Steady growth in this approach, on the other hand, might be incredibly beneficial when kids are learning about topics like human anatomy and physiology. The capacity to interpret and see knowledge such as brain function and nerve impulses, for instance, will aid in retention and a better understanding of human physiology. When examining the capacity of AR systems to allow several users to engage on the same framework, will aid in overcoming the isolation that these AR platforms may generate for learners. These AR-interfaced books are thought to provide a break for students from traditional textbooks’ stagnant and boring text-only learning material, making them an engaging tool for both students and instructors.

**Fig. 7:**
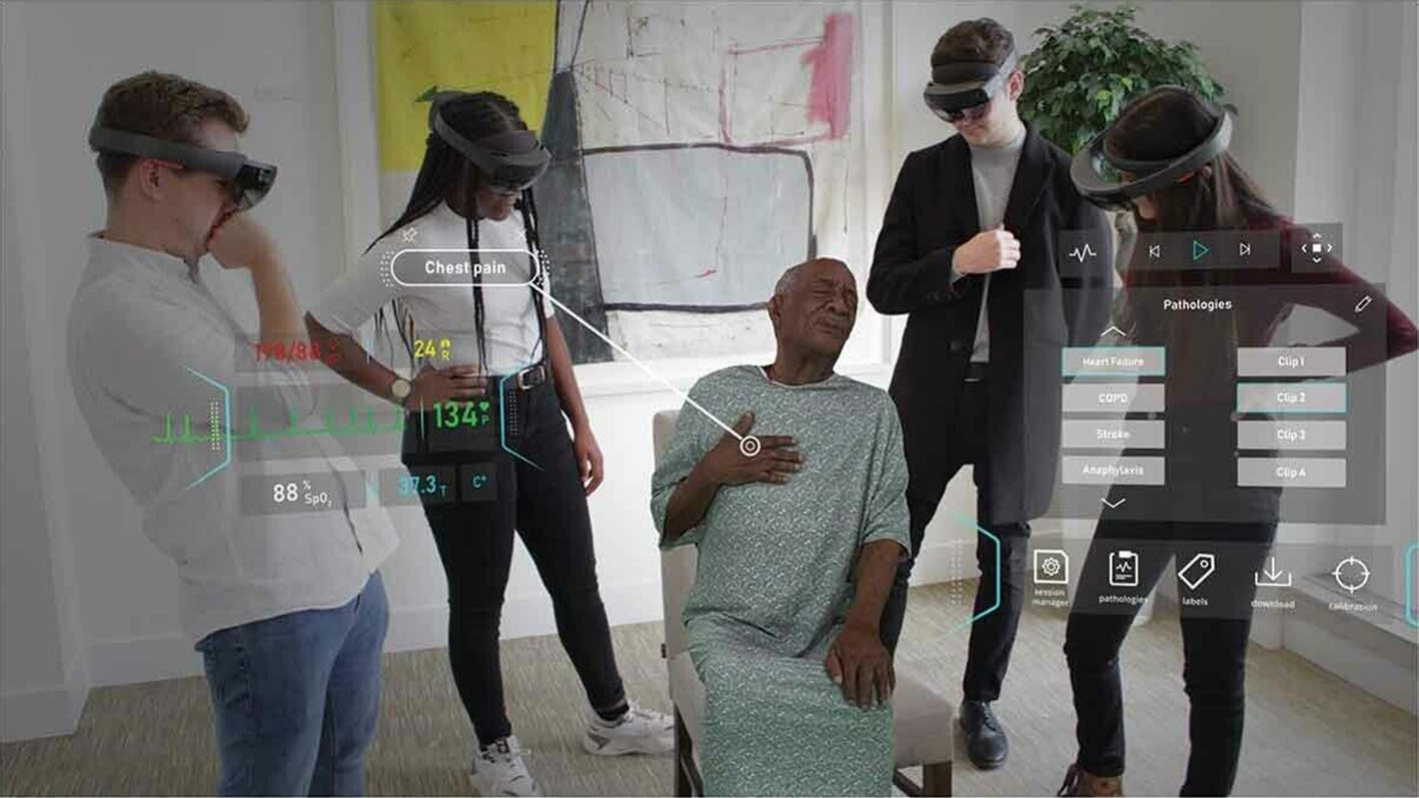
HoloPatient system [33]

Mobile learning and wearable technology are two new digital learning platforms with a lot of promise. Students’ own devices, such as smartphones, iPads, and tablet PCs, as well as wearable technology, such as smartwatches, might be used to provide AR-based learning software, making the use of this technology in medical education significantly more acceptable and affordable [40, 41]. The usage of Google Glass in anatomy classes and hospital rotations at the University of California, Irvine School of Medicine is one illustration of this approach [42]. Google Glass allows users to access course content and patient-related information without using their hands while also allowing them to converse by voice command [43]. Another wearable technology that might be employed in education is the usage of monitoring devices that can record a patient’s health and transfer it to the students’ smart devices, allowing them to identify an illness. The University of Michigan, for instance, is working on a vapor detector that will aid in the monitoring of patients with diabetes and lung illness [44]. A further option that might be effective in clinical school is the use of virtual patients and case scenarios during an issue-based instructional process (Fig. 7).

Figure 7 depicts a volumetric 3D video capture of a standardized patient seated in a chair being evaluated by a group of medical students using the HoloPatient system. Students can utilize the Microsoft HoloLens 2 to watch the patient and interact with the test results panel and real-time vital signs. In this instance, the patient is describing chest discomfort caused by myocardial infarction. Other key prospective possibilities for AR-based medical education include training programs for people with reading difficulties—a barrier to traditional textbook-based learning—and in remote learning, contexts to transfer users into a virtual realm wherever an Internet connection is available. Rendering AR technologies more affordable will be a major goal as the technology develops. The Medical Virtuality Lab, built by the University of Southern California’s Institute for Creative Technologies [45], is a famous example. This institute’s main goal is to bring together individuals and specialists from the film and gaming industries with computer and social scientists to develop and construct platforms for use in healthcare education and training.

The area of AR provides the potential for medical educators to construct a comprehensive and compelling curriculum that allows students to not only study but also experience educational information. Because of the disruption to traditional classroom teaching caused by COVID-19, technology-based instructional tools have been rapidly adopted throughout the world, underscoring the necessity of digital technology, such as AR, in ensuring that students’ learning is not impaired. Optimal and ongoing use of digital learning technologies has the ability to alter the medical education system, according to [46].

### XR in biomedicine

Although XR has many uses in industries like entertainment and education, its multiple uses in biomedicine open up revolutionary possibilities for both basic science and healthcare [47–50]. The authors in [51] concentrate on the process of turning biomedical findings into virtual things. By utilizing computer power and interactive analysis of real-world data like multidimensional imaging and macromolecular structures, using these virtual objects in XR boosts clinical studies and basic research. Several conversion pipelines and graphics rendering, which are facilitated by the immersive nature of XR, can be used to view data volumes. Since XR is interactive, it offers more scope for investigation and manipulation than regular monitor watching. They also go through several techniques for interacting with and visualizing biomedical data in XR applications.

Meanwhile, authors in [52] focused on novel affordance types that may influence XR-based training environments designed using HCI principles. Together with classification under the headings of visual and haptic affordance, new affordances are suggested. Moreover, the idea of dynamic affordance is put forth. Under the context of a surgical procedure known as condylar plating surgery, the effect of these affordances on cognition and skill acquisition is examined (which is performed to treat the fractures of the femur bone). Furthermore described is a genetic algorithm-based surgical planning strategy that can be applied to the training exercises. It also examined how the outcomes of assessment activities affected trainees’ learning of new skills and information while interacting with training settings.

Due to physiological adaptations, exposure to physical and psychological pressures, and restricted ability to provide medical treatment, spaceflight poses unique and substantial dangers to crew members’ health. Only a few studies have presented study findings on the applicability of XR technologies for enhancing space health, despite the fact that XR technologies are increasingly being embraced for training, real-time clinical care, and operational support in both terrestrial and aerospace contexts. To educate future research and development efforts on deploying XR technologies to improve space health and boost crew safety and performance, they also analyze the methodological and design characteristics of the previous studies [53].

The paper [54] describes the potential impact of XR on changing the healthcare sector, as well as its use cases, difficulties it presents, XR tools and techniques for smart healthcare, recent advancements of XR in intelligent healthcare services, and a discussion of potential advantages and potential future uses of XR techniques in the medical field. The paper [55] describes the gathering of spectrum data from a scene and mapping it onto a 2D image, hyperspectral, and multispectral imaging enable AR experiences. Short-wave infrared imaging is very effective for this imaging technique.

## AR and VR for rehabilitation

### Stroke rehabilitation during COVID-19

The shortage of rehabilitation experts is a global problem that is getting worse during COVID-19. For online learning, an AR Rehabilitation System was created. The virtual training was integrated into the participants’ regular care to lessen the effort of real instructors and alleviate staffing shortages. The contact rate in pandemics was also reduced as a result of this [56–59]. To see if AR Rehab-based virtual training can be successfully integrated into routine treatment in a real-world pandemic situation, the comprehensive program involved 129 patients from ten rehab facilities, with 39 of them being chosen for further evaluation.

As a result, the human–machine blended mode proved successful and efficient in reducing the work required by human rehabilitation specialists while still achieving the training objectives. Throughout pandemics, it alleviated the shortage of workers and lowered the interaction rate [60–62].

Authors in [63] described how some applications of XR-enabled platforms include post-stroke motor recovery and neuro-rehabilitation. As a result, this chapter focuses on mapping current research and development of immersive XR in the context of existing trivial applications and emerging nontrivial applications for patient recovery and wellness. To analyze recent academic and commercial advancements to expand immersive XR in the field of patient recovery and wellness, the authors also conduct a comprehensive study of the academic articles that are now available, international patent grants, and growing technical standards. During limb loss rehabilitation, limb loading improves skill transfer between augmented and physical reality tasks [64].

### Cancer and VR

A variety of unpleasant scenarios arise as a result of the diagnosis, accompanying therapies, and the more or less long-term consequences. As a result, it has been discovered that more than 10% of cancer patients have clinical signs of anxiety disorder, with the incidence rising to about 17.6% in patients who have had cancer for more than 2 years. VR is a therapeutic tool, but it also has a role in the oncology and cancer diagnostic coaching of professionals. VR and medical oncologies as a whole have a bright future [65].

VR is the most effective and widely utilized method for treating patients’ pain. Many oncology departments in affluent nations employ VR to assist their patients to cope with discomfort caused by the disease or following a biopsy. In [66], the authors reported that over half of hospital patients endure pain, with a fifth of the pain being regarded as “unbearable.” Pain has typically been treated with pharmaceutical interventions, such as opioids, which can have variable and unsatisfactory effects. Therapeutic VR has emerged as a viable non-pharmacological pain treatment option. [67, 68].

VR, according to one suggested mechanical hypothesis, operates as a distraction by activating the visual brain while engaging other senses, limiting the user’s processing of nociceptive stimuli. Because of the widespread availability of high-performance mobile computing, VR systems have shrunk in size and cost, allowing them to be used in common healthcare settings. To present, VR has been utilized in oncology to treat mental problems, reduce pain, promote treatment and rehabilitation, and divert sufferers during chemotherapy. Similarly, during operations like intravenous line insertion, VR decreases discomfort and provides a pleasant diversion [69].

VR is an outstanding tool to help adult patients and youngsters combat stress but also problems throughout chemotherapy sessions; it is a tech that will assist them to maintain morale throughout these sessions. As palliative treatment for cancer patients for worried patients, VR is recommended: Many scenarios in palliative care might cause anxiety (announcement of illness progression, care, chemotherapy treatments, radiation, and so on). The related aches wake up or intensify, which is often linked to anxiety episodes. Headphones might potentially help to calm these individuals by deflecting their focus past the pain and thereby reducing the discomfort dilemma.

**Fig. 8:**
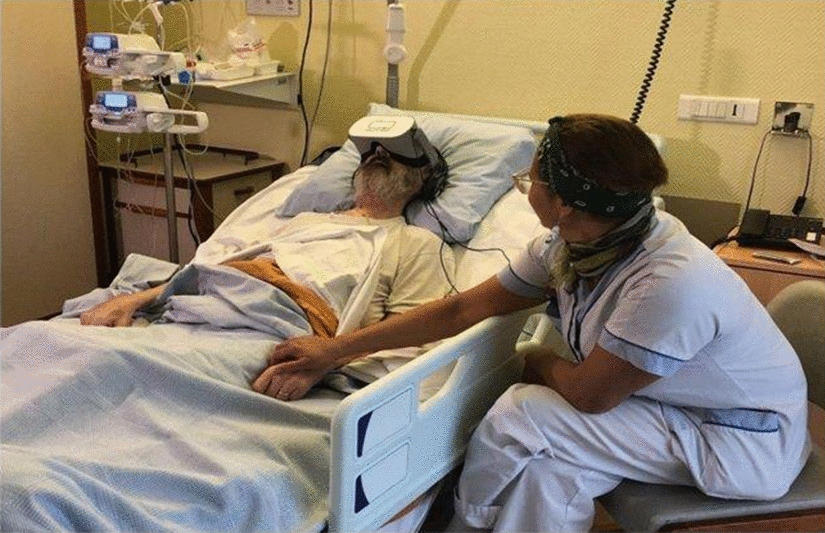
The VR headset decreases the anxiety of the intensive care patient [65]

As shown in Fig. 8, the VR headset can help prevent pain during certain treatments (installation of a peripheral venous route, Huber needle, indwelling catheter, blood gas analysis, and so on) and during the repair of complex dressings (dilapidated, very painful tumor wounds, sources of anxiety, and so on).

This device can be used in conjunction with standard treatments, such as anxiolytics, neuroleptics, and analgesics. It can also be of interest to people who have trouble following their medications. VR is altering the world around us, and healthcare teams are progressively considering its application in cancer. It is extremely interactive, versatile, personalized to the user, and suitable for people of all ages, genders, and health conditions. VR will likely affect the future of cancer care as technology advances and expenses decrease.

Through a comprehensive review, authors in [70] demonstrate how virtual reality technology can lessen cancer pain. Other metrics like weariness, sadness, anxiety, and stress were decreased in addition to pain intensity. Additionally, VR might make people feel more relaxed. The ability of VR to lessen pain may have positive effects on cancer pain treatment and patient comfort.

## Millimeter-wave and MIMO systems for AR and VR

Currently, improved holographic projection is supported by sixth-generation (6 G) networks thanks to terahertz (THz) bandwidths, incredibly low response time, and widespread device connection. Data are nonetheless sent across unsecured channels [71] between independent networks. As a result, an emerging research area is a partnership between Block Chain (BC) and 6 G in forthcoming AR/VR systems [72].

BC demonstrates tremendous promise for securing the AR/VR area. The following is a summary of the potential advantages of the 6 G and BC alliance in AR/VR:

- THz communication in sub-millimeter bands can be supported by 6 G networks with very low latency. The 6 G network provides virtualized service sets that provide holographic communications beyond physical barriers and support improved administration. It also enables huge communication in Internet-of-Things (IoT) networks, real-time XR for 3D imaging, autonomous automobiles, and industrial 4.0’s streamlined digital-twin processing.
- BC enables trusted decentralization across several nodes to guarantee huge data exchange among 6 G communication channels and to exploit confidence in AR/VR applications.
- Due to memory and processing constraints, the data are saved and transferred through a local central server for AR/VR assets that require quick image/video display and processing. The use of BC can improve data interchange security and collaboration.
- The central server may get overloaded owing to various asynchronous communication among AR/VR devices, since AR/VR-oriented apps are data and bandwidth-hungry. In this instance, BC can offer data decentralization.
- By leveraging AR/VR technology for law enforcement, public safety, and sensitive data exchange for military and combat purposes, BC may further boost cybersecurity. This greatly enhances the exchange of digital material and productive cooperation.
- BC can assist in the marketing of AR devices by developing a user-defined marketplace for decentralized storage and uploading of AR content. Additionally, the information may be kept and peer-reviewed using an interplanetary file system (IPFS), which enables authorized stakeholders to access records using an IPFS key. Hashed IPFS references are kept as external references in the BC ledger.
- Decentralization based in BC can assist developers and users in downloading and uploading material, as well as in building stores and marketplaces to facilitate the sale of AR/VR gadgets, for example; [73] Decentraland and [74] VibeHub.
- BC-based tokenization improves financial transactions in which AR devices serve as hardware and carry out peer-to-peer transactions on virtual currency [75].

Industry 4.0 and telemedicine are expected for one to gain the following 6 G services:

- The whole IoT control system would be strengthened by 6 G-TI services, which would enable a new, broad set of IIoT protocols to take the place of the outdated industrial Ethernet connection. The latter would provide physical processes with responsive automation.
- When combined with holographic communication, complicated machine learning, and deep learning models that would teach sensors and robotics to execute daily chores, 6 G would usher in a revolution in the IIoE that would be driven by immersive XR and digital-twin processes. The large training data would also be tightly coupled to provide extremely low-level device offloading latency thanks to sophisticated AI-based learning approaches.
- Through interactive and haptic AR services, healthcare 4.0 ecosystems would use 6 G to transform telemedicine and healthcare. Robots may, for instance, use responsive 6 G-based tactile Internet services to coordinate with distant clinicians to carry out telesurgical procedures. Additionally, with 6 G-based remote networks, VR services would enable clinicians to accurately analyze, magnify, and see 3D representations of bodily parts. This might make it possible for doctors to do medical treatments without being present in the clinics and to use surgical robots to perform difficult surgery.

To be able to automate the on-site monitoring process, this section shows a use-case of the industry 4.0 automation ecosystem that focuses on the automatic inspection of machines and processes. A handful of supply-chain stakeholders receive the analyzed data from the in-site monitoring systems once it has been processed by supported AI algorithms. We assume communication using 6 G-FeMBB service to make use of real-time automatic control to accommodate large bandwidth demands [76, 77]. BC is utilized at several supply-chain points to provide uniformity and timestamped chronology among users once the data have been supplied to the various supply-chain ecological stakeholders.

**Fig. 9:**
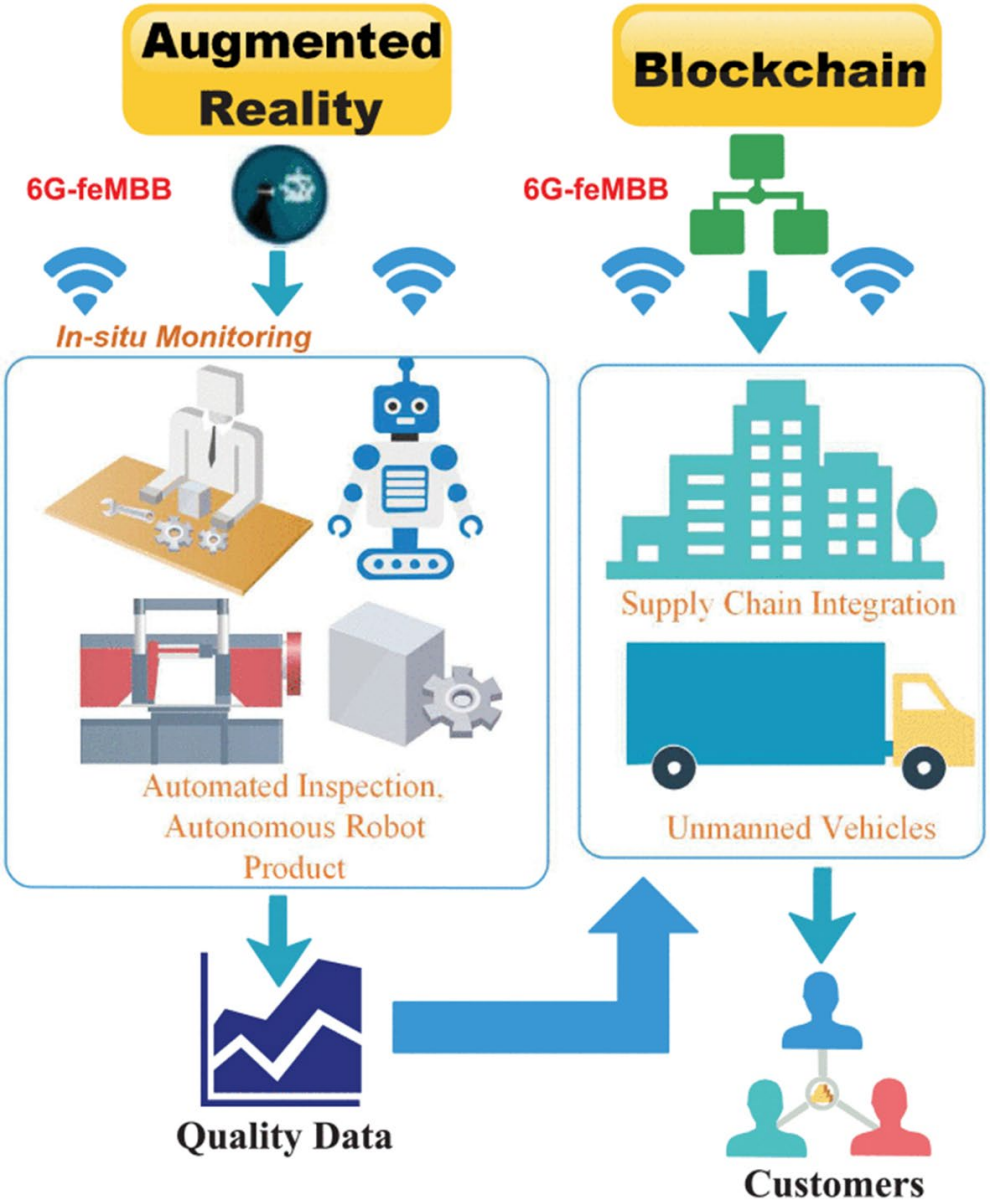
The decentralized system architecture in AR [71]

Figure 9 shows the architecture of a decentralized system along with AR and VR.

- Using AR in the Industry 4.0 process cycle: In this section, we will talk about how AR is a critical component of the automation cycle that underpins manufacturing, robotics, and automated quality inspection. Given the circumstances, industrial equipment is sensor-driven and interconnected to assist many activities. Robots are used at the on-site architecture to keep an eye on and physically examine the various procedures. Robotics decreases the demand for human involvement and is especially helpful in hazardous industries like the oil, gas, chemical, and petroleum sectors where boring and drilling activities pose a serious risk to human life. The robots gather sensor data, which is then combined and transferred to a cloud server for analysis.

Reinforcement learning is a good option to enhance robots’ learning, since it guarantees a reward–penalty-driven system, so that robots can learn and adapt to outside settings. Robotic motions may be observed visually, and the operation of the sensors and robots can be seen using AR-driven interactive controls. Network management is essential, since AR systems use a lot of bandwidth. Real-time automation is necessary for industrial processes for important applications, including bomb disposal, vehicle operation, heavy lifting, assembly pipelines, and many more. To guarantee accuracy and a stable network, 6 G-FeMBB networks can handle real-time bandwidth. Services like TI, muRLLC, and mMTC (for sensor-to-sensor communication) are favored for low latency.

- Data analytics: For Ground-Zero analytics, AR-gathered data like high-resolution videos and photographs of the daily process are employed. The data would be processed and the necessary visualizations would be provided via a back-end data visualization engine. Decentralization, encryption, and granting authorized individuals access to data are all guaranteed by BC.
- Supply-chain integration: In supply-chain ecosystems, BC ensures that every player in the chain, starting with the manufacturer, the supplier, logistics, warehouse storage, purchasers, and users, has access to chronological and timestamped points. Therefore, confidence is necessary at each crucial junction.

**Fig. 10:**
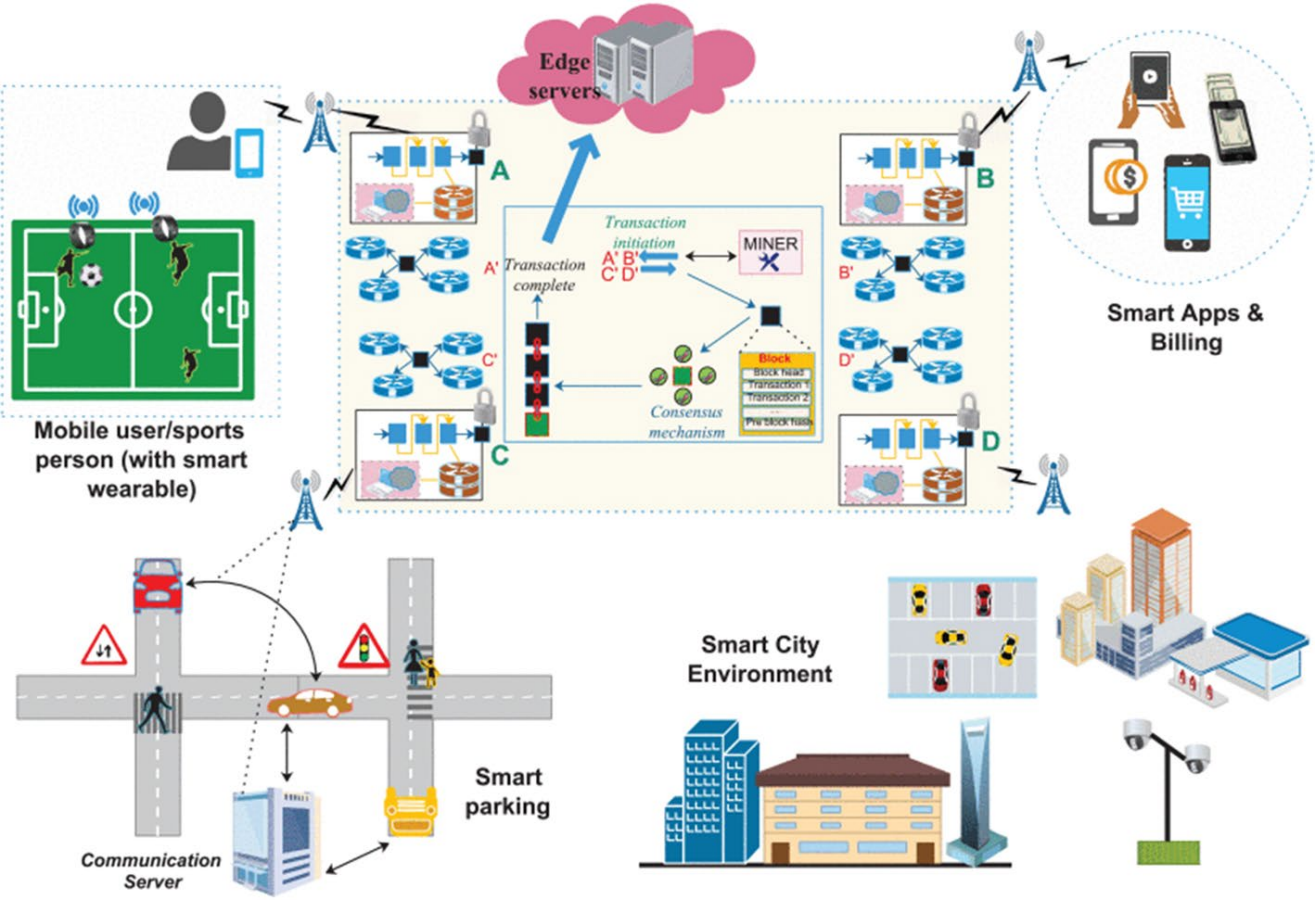
A proposed reference architecture of BC-based 6 G-envisioned massive IoT-supported AR/VR ecosystem [71]

Through connected SCs, BC facilitates automatic financial transfers between all stakeholders by leveraging the necessary confidence across all points in the supply-chain ecosystem.

As a result, manual involvement is diminished, middlemen and other third parties are cut out, and black marketing and product hoarding are cut out. This guarantees a seamless and transparent environment from beginning to finish. The traceability of mistakes is improved by BC, since it gives all users access to the same ledger state. Additionally, BC enhances the quality and proper access control of shared data, which lowers the cost of manufacturing. Since machine models can be easily examined through realistic 3D modeling, working machines and fault separation in AR and VR supply-chain systems become simple. Better output, lower costs, less downtime for machines, and enhanced profitability are the end results of AR/VR supporting supply chains.

A reference design for a smart city ecosystem is put out in [71] and supports a variety of IoT verticals, including automated factories, interactive XR, smart wearables, and smart automobiles. The essential elements of the enormous IoT-supported AR/VR ecosystems that BC-leveraged 6 G envisions are shown in Fig. 10.

## Results

**Fig. 11:**
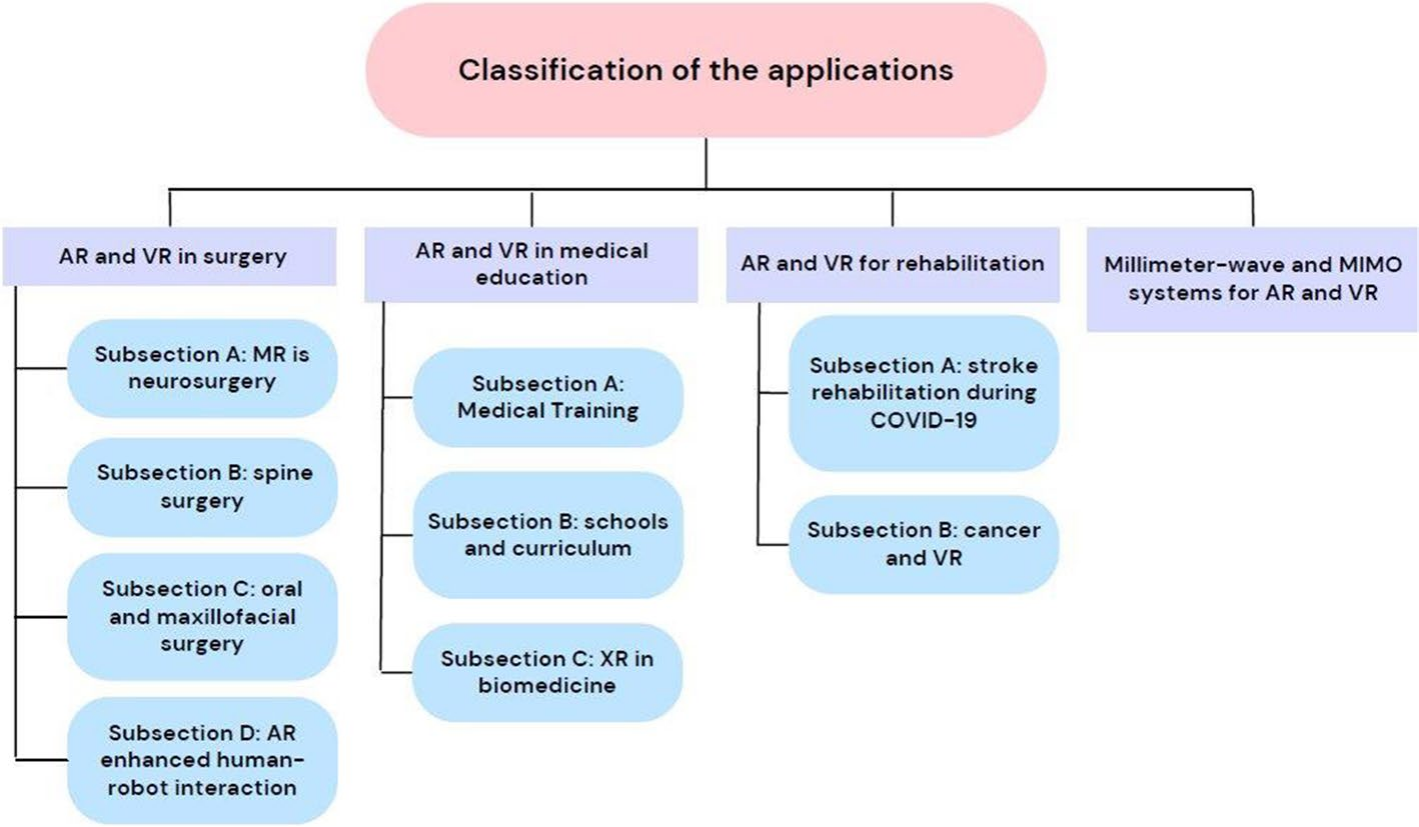
AR and VR in biomedical engineering: a review classification

Based on the inclusion requirements, 77 publications in total were chosen. Four distinct AR and/or VR applications groups could be differentiated, as shown in Fig. 11: AR and VR in surgery (*N* = 21) where (*N* = 3) cited in subsection A: MR in Neurosurgery, (*N* = 10) cited in subsection B: Spine Surgery, (*N* = 5) cited in subsection C: Oral and Maxillofacial Surgery, and (*N* = 3) in subsection D: AR-enhanced Human–Robot Interaction. Moreover, in the VR and AR in Medical Education (*N* = 30) as follows; (*N* = 11) cited in subsection A: Medical Training, (*N* = 10) cited in subsection B: Schools and Curriculum, and (*N* = 9) cited in subsection C: XR in Biomedicine.

Furthermore, AR and VR for Rehabilitation (*N* = 15) are as follows; (*N* = 9) cited in subsection A: Stroke Rehabilitation During COVID-19, and (*N* = 6) cited in subsection B: Cancer and VR. Millimeter-Wave and MIMO Systems for AR and VR (*N* = 7), where *N* is the number of cited studies. We found that the majority of research is devoted to medical training and education, with surgical or interventional applications coming in second. The research is mostly focused on rehabilitation, therapy, and clinical applications. Moreover, the application of XR in MIMO has been the subject of numerous research.

## Conclusion

There are plenty of other issues with AR and VR. Headaches, vertigo, and discomfort are common side effects of HMD, the chosen gear for usage by surgeons. AR and VR technologies have steep learning curves. Surgery professionals value tactile touch much, but current VR systems cannot accurately imitate it. The relative movement and/or distortion of soft tissue with regard to bones impairs skin marker monitoring systems. Nevertheless, with further technological advancement and more physician and engineer collaboration, virtually all of these constraints may be overcome.

There seems to be no denying that AR and VR have a bright future in several aspects of spine surgery, despite the fact that these technologies still have a diversity of drawbacks. These include their distinctive characteristics, cheap cost, and adaptability in merging with the other innovations. It is crucial to note that AR and VR have the potential to act as a bridge between a surgeon and an operating module, such as robots, by delivering information to the clinician and/or surgeon, students, trainees, or patients. The consequence is that AR and VR are organically linked with a variety of healthcare technologies. As these technologies evolve, new opportunities and uses for AR in healthcare may arise.

**Fig. 12:**
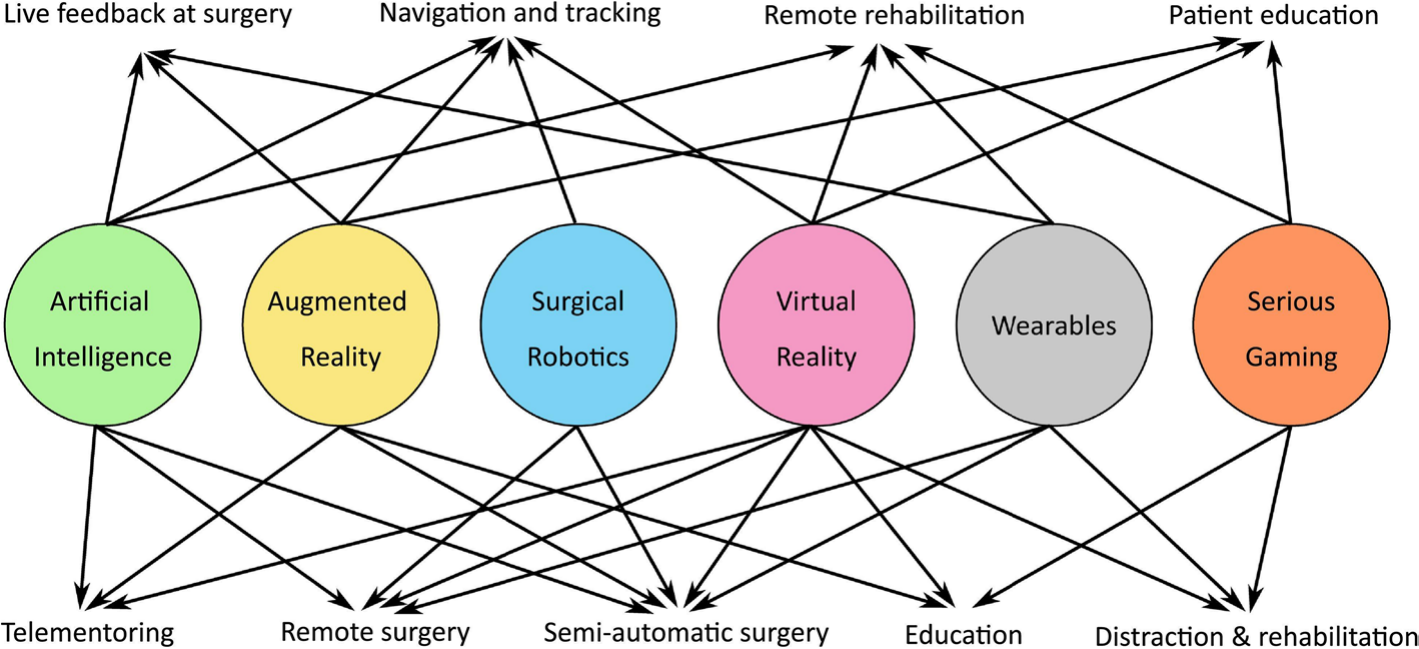
Technologies merged with AR and VR [17]

Illustrations of these possible integrations are shown in Fig. 12. The most popular AR and VR technology for patient education and students as well as providing pain relief for sufferers is gaming.

The development of remote therapeutic exercise settings where the doctor interacts and monitors the patient in a virtual world may be made possible by the integration of AI, wearable sensors, gaming, and AR and VR. AI and AR may be used with robot-assisted navigation systems to produce user-friendly, quick, and accurate navigation systems. To give surgeons real-time input during surgery, AI, wearables, and AR can be coupled. Telementoring and its connection with wearables can be advanced by combining AR, VR, and AI, and surgical robotics can enable remote and semi-automatic procedures.

Within a few decades, It is probable that surgeons would consult with patients, treat them, educate them, and do surgery while sporting AR glasses. AR is set to significantly influence all areas of orthopedics. The next wave of spine surgeons will undergo appropriate training in XR. Being a leader in orthopedics, spine surgery ought to be eager to acquire these innovative techniques. In the ensuing 10 decades, we will be able to combine the efforts of physicians, engineers, and game designers to significantly enhance every aspect of spine surgery.

## Data Availability

All data and materials included in this study are available online and have open-access.

## Abbreviations

XR: Extended reality
MR: Mixed reality
VR: Virtual reality
CT: Computed tomography
HMD: Head-mounted display
3D: Three-dimensional
ARSN: Augmented reality surgical navigation
VPAR: Virtual protractor with an augmented reality
AI: Artificial intelligence
MRI: Magnetic resonance imaging
OCMS: Cranio-maxillofacial surgery
IGS: Integrated global services
HRI: Human–robot interaction
ARiSE: AR in Surgery and Education
6 G: Sixth generation
THz: Terahertz
BC: Block chain
IoT: Internet-of-Things
IPFS: Interplanetary file system
